# Whole-Body Teleoperation Control of Dual-Arm Robot Using Sensor Fusion

**DOI:** 10.3390/biomimetics8080591

**Published:** 2023-12-05

**Authors:** Feilong Wang, Furong Chen, Yanling Dong, Qi Yong, Xiaolong Yang, Long Zheng, Xinming Zhang, Hang Su

**Affiliations:** 1 College of Mechanical and Electrical Engineering, Changchun University of Science and Technology, Changchun 130022, China; feilong_wang0707@163.com (F.W.); chenfurong0721@163.com (F.C.); yangxiaolong0227@163.com (X.Y.); 2 Key Laboratory of Bionic Engineering, Ministry of Education, Jilin University, Changchun 130022, China; 3 Weihai Institute for Bionics, Jilin University, Weihai 264402, China; 4 School of Foreign Languages, Shandong University, Jinan 250100, China; yanling.dong2@gmail.com; 5 ESIEE Paris, 93160 Noisy-le-Grand, France; qi.yong@edu.esiee.fr

**Keywords:** humanoid arm, sensor fusion, teleoperation

## Abstract

As human–robot interaction and teleoperation technologies advance, anthropomorphic control of humanoid arms has garnered increasing attention. However, accurately translating sensor-detected arm motions to the multi-degree freedom of a humanoid robotic arm is challenging, primarily due to occlusion issues with single-sensor setups, which reduce recognition accuracy. To overcome this problem, we propose a human-like arm control strategy based on multi-sensor fusion. We defined the finger bending angle to represent finger posture and employed a depth camera to capture arm movement. Consequently, we developed an arm movement tracking system and achieved anthropomorphic control of the imitation human arm. Finally, we verified our proposed method’s effectiveness through a series of experiments, evaluating the system’s robustness and real-time performance. The experimental results show that this control strategy can control the motion of the humanoid arm stably, and maintain a high recognition accuracy in the face of complex situations such as occlusion.

## 1. Introduction

In recent years, the rise in anthropomorphic robots has permeated various sectors with the growing trend of robot humanization and human–robot societal integration [1], including medicine [2], education [3], industry, and human–robot interaction [4]. The humanoid robotic arm, a pivotal component of these robots, has significantly propelled advancements in robotic technology. By incorporating anthropomorphic motion control in teleoperation [5], it becomes more intuitive and convenient to control these robots, further advancing the field.

The journey toward enhanced teleoperation control of humanoid robotic arms has been significantly marked by several key progress areas: one of the pivotal areas of progress lies in sensor fusion technologies. In recent years, sensors have undergone transformative developments, leading to substantial improvements in teleoperation control precision and efficiency. Visual sensors, such as high-resolution cameras and depth sensors [6], have experienced significant enhancements in both accuracy and speed [7]. These improvements enable robots to capture and process visual information with unprecedented fidelity. The introduction of new methods enables robots to perceive objects effectively [8,9]. Furthermore, force and tactile sensors have evolved to become more sensitive and reliable, endowing robots with the ability to interact with their environments in a more nuanced manner [10,11]. The integration of inertial measurement units (IMUs) has raised the bar in terms of precision and latency, significantly enhancing robot state estimation [12]. Collectively, these strides in sensor technology have paved the way for more effective teleoperation by providing robots with a richer and more accurate understanding of their surroundings.

Another transformative dimension of progress has been the fusion of machine learning techniques with sensor data. This fusion has considerably enriched the perceptual and decision-making abilities of robots. Machine learning models, such as convolutional neural networks (CNNs) and recurrent neural networks (RNNs), have excelled in feature extraction and real-time data processing. Reinforcement learning algorithms have empowered robots to adapt dynamically to ever-changing and uncertain environments, thereby elevating their autonomy and capacity to comprehend complex surroundings. By harnessing the power of machine learning and adaptive learning [13,14] in sensor fusion, robots have become more adept at interpreting sensory information, making informed decisions, and executing tasks with precision and efficiency.

In tandem with these advancements, real-time feedback mechanisms and sophisticated haptic interfaces have revolutionized teleoperators’ ability to control robots. These developments provide teleoperators with immediate sensory feedback from the robot’s sensors, enhancing their situational awareness, and fine-grained control over robotic actions. Operators can now perceive and respond to the robot’s interactions with the environment in real-time, resulting in a more intuitive and responsive teleoperation experience.

Another avenue of exploration has been the integration of various sensor modalities [15,16], creating comprehensive perception systems that can handle a wide spectrum of tasks and environments. Researchers have successfully combined visual sensors with force/tactile sensors [9,10], IMUs, and proprioceptive sensors. This multi-modal approach [17] has endowed robots with a holistic understanding of their surroundings, making teleoperation more versatile than ever before. Additionally, this integrated sensing system is not only applicable to upper limb motion perception but also equally effective for lower limb applications. In the fields of lower limb rehabilitation, sports assistance [18], and lower limb prosthetics [19], sensor fusion systems have also been widely utilized.

Complementing these technological advancements, a substantial body of related works has further enriched the teleoperation landscape [20]. These works encompass sensor fusion strategies for enhancing operator control and situational awareness, machine learning-based approaches that adaptively fuse sensor data for more efficient teleoperation [21], haptic feedback systems that provide operators with tangible sensations of touch and force feedback, as well as investigations into human–robot interaction during teleoperation [22], aiming to create intuitive interfaces that bridge the gap between human intent and robotic action. Additionally, researchers have prioritized safety in dual-arm robot control by developing redundancy mechanisms [23] and safety protocols to handle sensor failures and unexpected events, particularly in contexts where human lives or valuable assets are at stake.

Collectively, these remarkable strides in sensor technology, machine learning, real-time feedback, and multi-modal sensor integration, alongside the wealth of related works, have fueled the ongoing development of whole-body teleoperation control for dual-arm robots using sensor fusion.

However, achieving remote real-time motion control of humanoid robotic arms is a challenging and critical research area [24]. It aims to overcome challenges such as geographical distance and time delays, ensuring that operators can control robot arms in real time from remote locations. This necessitates highly sensitive sensor technology [25,26], reliable communication networks, and intelligent control systems. Furthermore, accurate perception of the posture and position of human arms is also crucial. And one of the main difficulties is the precise perception of hand motion [27], which requires a robust and reliable sensing system. Many wearable devices have been extensively validated for their effectiveness in human-computer interfaces, robot control, and exoskeleton intent control research [28,29,30,31]. Wearable data gloves have also been proven to enable continuous control of bionic hands, and numerous related studies have confirmed the stability and reliability of this control method [32,33]. However, for this research, I chose to use non-contact sensors such as Leap Motion sensors and Microsoft Kinect sensors [34,35,36,37,38,39] mainly because these sensors offer convenience, cost-effectiveness, and high resolution [40,41]. It is important to note that these sensors are only used indoors. While both sensors can track hand movements, research by Kim et al. shows that the Leap Motion sensor is more accurate than the Kinect [42]. Leap Motion can provide high-precision tracking of hand and finger movements and provides a rich software interface for posture and gesture recognition. It has the advantages of robustness, portability, and high precision. However, the Kinect depth camera does a good job of capturing arm motion.

Though the Leap Motion controller has limitations, especially in terms of accuracy and occlusion, multi-sensor fusion has emerged as a solution to ensure consistent data flow. Researchers have combined various sensors for precise hand movement perception. For example, Marin et al. made the first attempt to detect gestures by fusing data from Leap Motion and Kinect [43]. Eider C. P. Silva et al. used sensor fusion technology to fuse data from the Leap Motion and Myo Armband to create a 3D virtual simulation of the arm’s movement, including the forearm, hand, and fingers, in real time [44].

Compared to other sensor fusion strategies, the multi-Leap Motion fusion strategy offers significant advantages in terms of data and algorithms. While each sensor may have inherent errors and limitations, the fusion of data from multiple sensors of the same type allows for a substantial reduction in errors and an enhancement of system reliability. In addition, a reasonable spatial layout can also compensate for occlusion issues that arise when using a single Leap Motion controller [45]. And when it comes to complex motion tracking, the use of multiple sensors can more accurately capture and identify various poses and movements. The multi-sensor system can provide more data perspectives. In this paper, three leap motion sensors are adopted, and the position and attitude angle of the sensors can be adjusted arbitrarily. Through reasonable spatial layout, the sensors can effectively cover each other’s blind areas and provide continuous tracking when the hand rotates, so as to achieve all-round perception of the hand motion. However, For motion perception of the arm, using the Kinect depth camera is a better solution.

This paper introduces an innovative control strategy for anthropomorphic arms based on sensor fusion. Our research primarily focuses on the following key aspects:

- **Mapping human finger motion to a 6DOF bionic hand:** We present a novel approach that maps human finger motion models onto a 6-degree-of-freedom (6DOF) bionic hand. This mapping allows for direct control of the bionic hand by defining finger-bending angles.
- **Accurate hand motion perception:** To achieve precise perception of hand motion, we leverage multiple Leap Motion sensors. Our method ensures precise control of the bionic hand, even in scenarios where sensors may be obstructed or fall out of their field of view.
- **Pressure sensor-based force control:** Safety is of utmost importance in our control strategy. The fingertips of the bionic hand are equipped with pressure sensors, and we propose an innovative pressure sensor-based force control strategy to ensure the safety of control and operation.
- **Arm control using Kinect depth camera:** We extend our control strategy to encompass the motion and pose of the human arm. By utilizing a Kinect depth camera, we can perceive arm motion, enabling control of the anthropomorphic arm’s movements within a specific spatial range.
- **Data fusion with Kalman filtering:** To overcome the limitations of individual sensors, we employ the Kalman filtering algorithm for data fusion. This enhances the stability and reliability of our system.

## 2. Methodology

### 2.1. Construction of Arm Motion Tracking System

#### 2.1.1. Anthropomorphic Dual-Arm Robot with Bionic Hands

**Figure 1:**
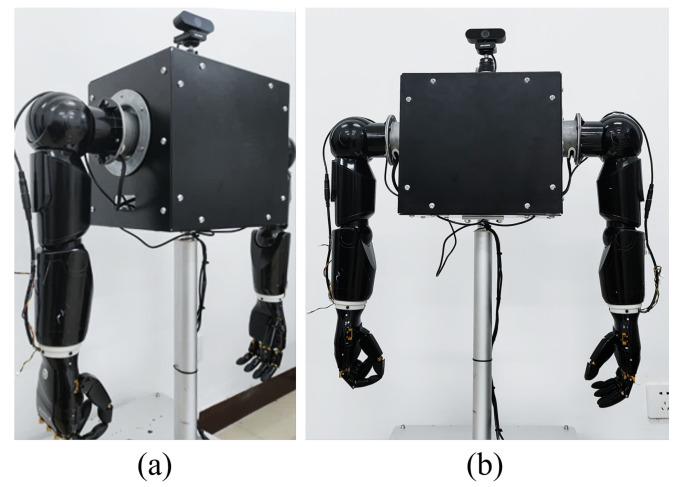
Physical drawing of an anthropomorphic dual-arm robot. (**a**) Left view; (**b**) front view.

In our previous work, we developed an anthropomorphic dual-arm robot with a bionic hand, which contains two humanoid arms with 5 DOF each, including 2-shoulder DOF, 2-elbow DOF, and 1-wrist DOF [46]. The two arms are mainly responsible for the movement of the end-effector within a certain space, which is a bionic hand with six degrees of freedom, including two degrees for the thumb and one degree for each of the other four fingers. In addition, each fingertip is equipped with a tactile sensor, which consists of a multi-point array of pressure sensors, with the single-point pressure sensor measuring a range from 0 to 5N. The bionic hand has a fast response speed and supports a variety of communication methods, such as WIFI and Bluetooth. By working with two arms, the humanoid two-armed robot can perform a series of delicate and complex operational tasks. The anthropomorphic dual-arm robot is shown in Figure 1.

#### 2.1.2. Hardware System

**Figure 2:**
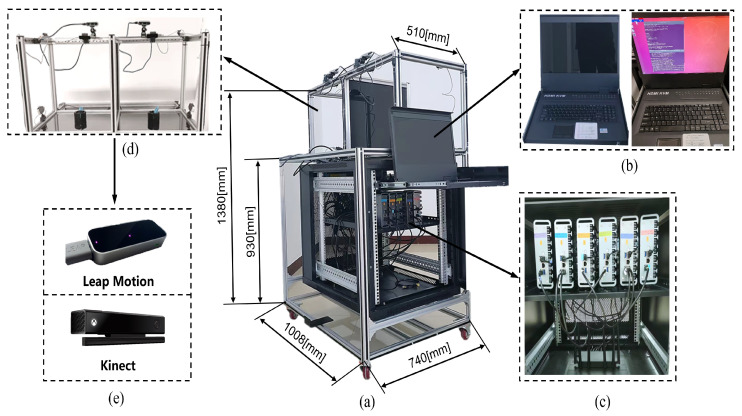
Arm tracking system construction. (**a**) System overview; (**b**) integrated display system; (**c**) data processing system; (**d**) signal acquisition section; (**e**) Leap Motion controller and Kinect depth camera.

This paper constructed an arm movement tracking system based on multi-sensor fusion. The system consists of a cabinet, six Leap Motion sensors, six microcomputers, a Kinect depth camera, a KVM switcher, and a router. The main structure of the system is shown in Figure 2. In this system, there are two RealSense cameras and two Myo armbands, which are not used in this article, so it is not covered.

**Figure 3:**
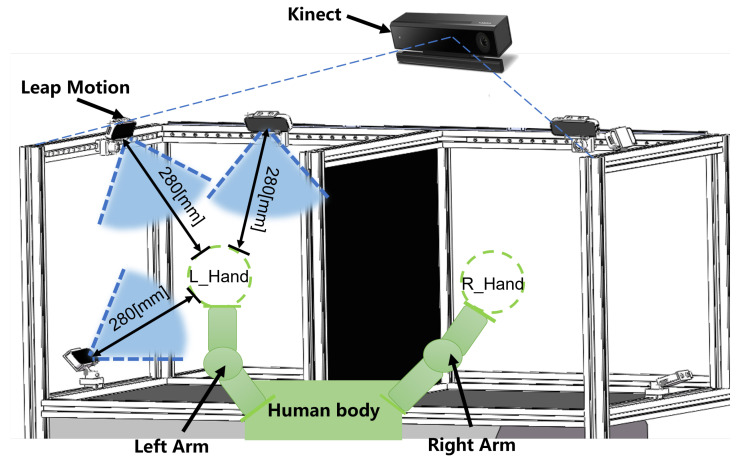
Spatial position diagram of arms and sensors.

To provide a clearer depiction of the overall framework and structure of the motion capture system, we have also included a Figure (Figure 3).

#### 2.1.3. Software System

The system employs ROS as the underlying communication framework and establishes connections with six miniaturized computers. The choice of ROS as the communication framework is based on its wide adoption and validation in the field of robotics [47,48,49]. It provides robust tools and libraries for sensor data acquisition, processing, distribution, and the implementation of robot control. Furthermore, ROS is characterized by its open-source nature, enabling highly customizable development. It supports multiple programming languages, including C++ and Python, making it more flexible and user-friendly. We use a multi-machine communication mechanism, so that each computer only needs to run a specific ROS node to obtain data from the six Leap Motion sensors. However, the use of multi-machine communication does not guarantee that the times of all devices are completely synchronized, so we use a special time synchronization tool—network time protocol (NTP)—to help devices synchronize their system time in the network to ensure that their time is highly consistent. In order to ensure the stability of the sampling period and the real-time performance of the system, in addition to using NTP to synchronize the system clock, we also use the ROS time management tool to control the time interval of message release when designing ROS nodes. By subscribing to topics published by miniaturized computers, an external computer can receive data from all the Leap Motion sensors. Additionally, we also retrieve data from the Kinect depth camera through the external computer. Ultimately, all sensor data will be comprehensively processed on the external computer.

**Figure 4:**
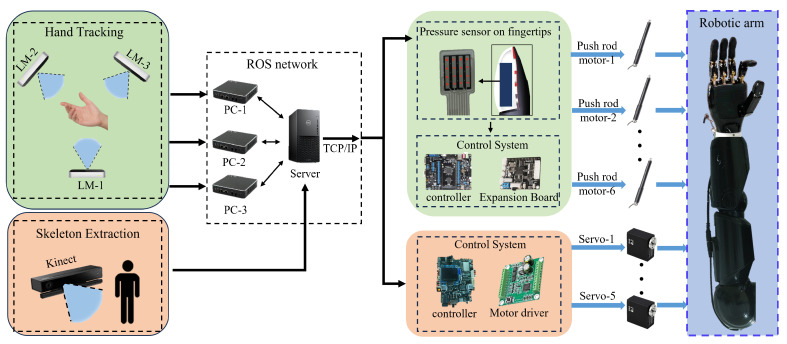
Software system description diagram based on a single arm.

It is worth noting that we have utilized ROS-based software development techniques to achieve real-time monitoring and tracking of arm movements, enabling operators to control the humanoid arm in real time for more intricate tasks. In summary, this system effectively leverages advanced sensor fusion technology and ROS-based software development techniques, combined with filtering algorithms, to achieve highly precise perception of arm movements and real-time control of the humanoid mechanical arm. In terms of hardware systems, we use multiple Leap Motion sensors to capture both left- and right-hand movements, and the sensors have the same spatial layout. In addition, we use the Kinect depth camera to sense the movements of the human arm. Therefore, at the level of motion capture and system architecture, there is no difference between the left and right arms. For this reason, we chose a unilateral arm for the analysis of the software system. The specific software system diagram is shown in Figure 4.

### 2.2. Mapping from Human Arm Kinematics to the Anthropomorphic Arm

#### 2.2.1. Mapping Strategy Statement

**Figure 5:**
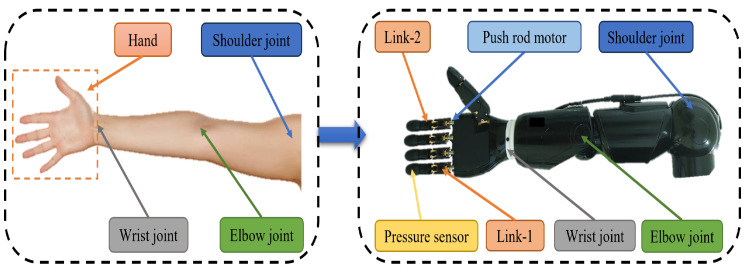
The correspondence between a human arm and an anthropomorphic arm.

In this paper, we propose a strategy to achieve human-like control of humanoid arms. Figure 5 illustrates the correspondence between the human arm and the anthropomorphic arm. In the bionic hand, the flexion and extension of each finger are controlled by a single push rod motor and several linkages. The bionic hand has a total of 6 degrees of freedom, which is significantly fewer than the degrees of freedom in the human hand. This shows that we cannot directly use the detailed angles of each finger bone to control the bionic hand. The number of degrees of freedom of the anthropomorphic arm and the human arm is not much different, and the angle of the human arm joint can be mapped to the humanoid arm by kinematic mapping.

In order to achieve accurate and stable control of the underactuated bionic hand and avoid the interference of redundant information, a new kinematic variable, finger bending angle, is introduced. The finger bending angle mainly reflects the trend of finger bending. We mapped the finger bending angle to the push rod motor of the bionic hand to control the expansion and contraction of the push rod motor, thus controlling the flexion and extension of the bionic hand. In order to obtain the motion data of the skeletal points of the hand, we used the leap motion controller and established a coordinate system on the hand. Through coordinate transformation, the bone point data obtained by the leap motion controller is uniformly converted to the hand coordinate system. Because the hand coordinate system is independent of the bone tracking coordinate system, it is treated as a common coordinate system, which allows the leap motion controller to be placed in any position without calibration.

To achieve control of the humanoid arm, we employ Kinect to recognize and extract three-dimensional coordinates of the human body’s skeletal structure. Through algorithmic calculations, we determine the angles of various joints in the human arm and subsequently map them onto the corresponding servos of the humanoid arm for control.

#### 2.2.2. Definition of arm Kinematic Mapping

**Figure 6:**
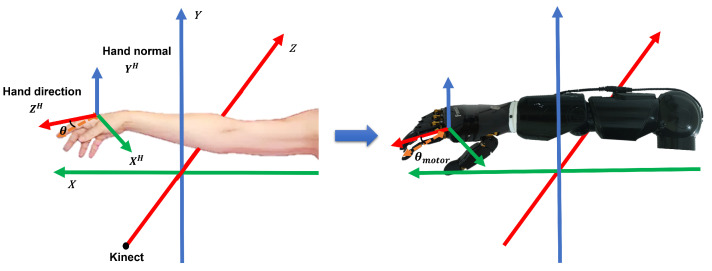
The hand motion is transferred to the underactuated bionic hand through the coordinate system of hand motion. The skeleton point data captured by the Leap Motion controller was converted into the hand coordinate system to calculate the bending angle. Establishing a spatial coordinate system for capturing arm movements with the Kinect depth camera as the origin, we can calculate joint angles to drive the humanoid arm.

Figure 6 illustrates the coordinate framework of the arm, where the $X\in R^{3}$, $Y\in R^{3}$, and $Z\in R^{3}$ axes are the space rectangular coordinate system established with Kinect as the coordinate origin. Kinematic mapping for the arm, taking the elbow joint’s degree of freedom as an example, we calculate the angle between the vector from the elbow to the wrist and the vector from the elbow to the shoulder. This angle is then mapped onto the corresponding servo of the humanoid arm, enabling remote control of the humanoid arm’s elbow joint in an anthropomorphic manner. The specific calculation formula is as follows [50]:

(1) $$\beta_{\mathrm{elbow}}=\arccos\frac{a\cdot b}{\left|a\right|\cdot \left|b\right|}$$

where *a* represents the vector from elbow to wrist, and *b* represents the vector from elbow to shoulder.

In Figure 6, $Z^{H}\in R^{3}$ represents the direction vector of the hand and $Y^{H}\in R^{3}$ represents the normal vector of the palm plane, and $X^{H}=hand\;direction\times hand\;normal\in R^{3}$. We employ the newly defined kinematic variable, “finger bending angle”, to describe the movement trend of the fingers for controlling the underactuated bionic hand. The angle between the pointing vector of each finger and the horizontal plane of the palm determines the finger’s bending angle. To achieve this, we need to redefine the pointing vector of the fingers, where the starting point is the distal end of the proximal phalanx, and the ending point is the starting end of the distal phalanx. Taking the index finger as an example, the calculation formula is as follows:

(2) $$\theta_{index}=\arcsin\frac{v_{index}\cdot Y^{H}}{\left|v_{index}\right|\cdot \left|Y^{H}\right|}$$

where $v_{index}\in R^{3}$ represent the pointing vectors corresponding to each finger. This methodology can be applied to determine the bending angle of all five fingers, including the thumb, index, middle, ring, and pinky fingers.

It is worth noting that the underactuated bionic hand’s thumb possesses two degrees of freedom. Equation (2) solely determines one of these degrees of freedom, specifically, the bending angle of the thumb. The other degree of freedom corresponds to the thumb’s abduction and adduction, which can be quantified by the angle between the thumb’s pointing vector and $X^{H}\in R^{3}$. The calculation formula is as follows:

(3) $$\alpha_{thumb}=\arccos\frac{v_{thumb}\cdot X^{H}}{\left|v_{thumb}\right|\cdot \left|X^{H}\right|}$$

where $\alpha_{thumb}$ represents the angle of thumb abduction and adduction of the control underactuated bionic hand, and $v_{thumb}$ represents the pointing vector of the thumb. Since the thumb has three phalanges, this vector starts at the end of the metacarpal and points to the beginning of the distal phalanges.

### 2.3. Multi-Sensor Data Fusion and Control Strategy

#### 2.3.1. Sensor Fusion Framework

We use the Kalman filter to estimate the angle of each joint of the arm and hand to obtain more stable and reliable angle data. Data for each joint of the hand comes from multiple Leap Motion sensors. Taking the hand as an example, the state space model of the hand can be summarized as [51]

(4) $$\begin{array}{cc} x_{k} & =\Phi_{k}x_{k-1}+\Gamma_{k}u_{k}+w_{k}\\ z_{k} & =H_{k}x_{k}+v_{k}, \end{array}$$

where $x_{k}\in R^{n}$ is a state vector, which contains information describing hand posture and movement, mainly including hand joint angles and other signal sources related to control and measurement. $z_{k}\in R^{m}$ is a measurement vector that mainly contains measurements from multiple Leap Motion sensors. $u_{k}\in R^{l}$ is the input vector that contains the actual value of the control input. $\Phi_{k}\in R^{n\times n}$, $H_{k}\in R^{m\times n}$, and $\Gamma_{k}\in R^{n\times l}$ represent the state transition model, observation model, and control input model, respectively. The state transition model is used to describe the state evolution of the system between time step *k* and time step k−1. It defines the evolution of the state vector. The observation model describes the relationship between the state vector $x_{k}$ and the measurement vector $z_{k}$, and maps the state vector to the measurement space. The control input model represents the influence of the control input on the state variable. $w_{k}$ and $v_{k}$ represent process noise and measurement noise, respectively. Among them, process noise represents the uncertainty or interference of the system model, including noise components related to the dynamics of the system. Measurement noise represents errors or noise components in the measurement process. The two are independent of each other.

(5) $$\left.w_{k}\right.∼N\left(0,Q\right)$$

(6) $$\left.v_{k}\right.∼N\left(0,R\right)$$

where *Q* is the covariance matrix of process noise, which represents the uncertainty or noise in the evolution of the state of the system between time steps. In general, the higher the value of *Q*, the higher the uncertainty of the system state change. *R* is the covariance matrix of measurement noise, which represents the uncertainty or noise from the sensor measurement. In general, the larger the value of *R*, the higher the uncertainty of the sensor measurement. In this article, both *Q* and *R* are obtained by manual adjustment to ensure that the model accurately reflects the properties of the actual system. Furthermore, for the analysis of hand motion, the control input $u_{k}$ generated in the muscular system is considered unknown. Alternatively, the hand can be modeled as a rigid body in space. The linearized motion model of a rigid body in space can be written as [52]

(7) $$\begin{array}{cc} \; & x_{k}=x_{k-1}+T_{s}{\dot{x}}_{k}+T_{s}^{2}/2{\ddot{x}}_{k}\\ \; & {\dot{x}}_{k}={\dot{x}}_{k-1}+T_{s}{\ddot{x}}_{k}, \end{array}$$

where $x_{k}$ and $x_{k-1}$ represent the motion sequences of *k* and k−1, respectively. $T_{s}$ is the time interval, indicating the time difference between two adjacent time steps. ${\dot{x}}_{k}$ and ${\dot{x}}_{k-1}$ represent the speed of the system at time step *k* and k−1, respectively. One way to model the acceleration term ${\ddot{x}}_{k}$ is to consider it as a form of process noise. We chose $x_{k}$ as the state variable for the system model. Therefore, the system model can be expressed as

(8) $$x_{k}=\Phi_{k}x_{k-1}+\Gamma_{k}w_{k}.$$

It is important to note that $\Gamma_{k}$ no longer represents the control input model but rather a model describing the influence of process noise.

#### 2.3.2. Fusion Algorithm


(9) $$\begin{array}{cc} \; & {\hat{x}}_{k|k-1}=\Phi_{k}{\hat{x}}_{k-1|k-1}\\ \; & P_{k|k-1}=\Phi_{k}P_{k-1|k-1}\Phi_{k}^{T}+\Gamma_{k}Q_{k}\Gamma_{k}^{T}. \end{array}$$

Kalman filter is a recursive filter, where ${\hat{x}}_{k|k-1}$ is the value of the next state estimated in the first stage of the Kalman filter (prediction stage), representing the estimated state at the time step *k*, based on the prediction of the system model. $P_{k|k-1}$ is the estimated state covariance matrix in the prediction stage, representing the uncertainty of the estimated state.

In each cycle, the process is updated iteratively

(10) $${\hat{x}}_{k|k}={\hat{x}}_{k|k-1}+K_{k}{\tilde{y}}_{k}$$

where ${\hat{x}}_{k|k}$ is the estimated state value in the second stage of Kalman filtering (the update stage), taking into account the impact of the measurement results.

$K_{k}$ is the Kalman gain that is used to incorporate the measurement results into the state estimate. ${\tilde{y}}_{k}$ is the measurement residual that represents the difference between the actual measured value and the predicted value, $S_{k}$ is the variance matrix of the measurement residuals, representing the uncertainty of the measurement residuals, usually consisting of the measurement noise and the uncertainty of the system model. They are mathematically defined as follows [39]

(11) $$\begin{array}{cc} K_{k} & =P_{k|k-1}H_{k}^{T}S_{k}^{-1}\\ {\tilde{y}}_{k} & =z_{k}-H_{k}{\hat{x}}_{k|k-1}\\ S_{k} & =H_{k}P_{k|k-1}H_{k}^{T}+R_{k}. \end{array}$$

An updated estimate of the covariance matrix

(12) $$\begin{array}{cc} {\hat{P}}_{k|k} & =\left(I-K_{k}H_{k}\right)P_{k|k-1}\\ Q & =\left[\begin{array}{ccc} \sigma_{x}^{2} & 0 & 0\\ 0 & \sigma_{y}^{2} & 0\\ 0 & 0 & \sigma_{z}^{2} \end{array}\right] \end{array}$$

where ${\hat{P}}_{k|k}$ is the estimated state covariance matrix in the update stage of the Kalman filter, representing the uncertainty of the state estimation after the update. *I* is the identity matrix. *Q* is the covariance matrix of the process noise, where $\sigma_{x}^{2}$, $\sigma_{y}^{2}$, and $\sigma_{z}^{2}$ represent the uncertainty or noise of the state variable in the direction of *x*, *y*, and *z*, respectively.

#### 2.3.3. Adaptive Strategy

Due to occlusion, not all sensors can detect hands correctly in most cases. Therefore, we define a function to calculate the confidence of the tracking data. We limit the range of confidence to be between 0 and 1. When the normal of the palm forms a 90-degree angle to the *Y*-axis of the Leap Motion controller, we set the confidence level to 0. In addition, when the normal of the palm forms a 0 or 180-degree angle to the *Y*-axis of the Leap Motion controller, we set the confidence level to 1. Angles over 180 degrees or less than 0 degrees are mapped into this range. The mathematical expression of the function is as follows [53]

(13) $$confidence=\frac{4}{\pi^{2}}\alpha^{2}-\frac{4}{\pi }\alpha +1$$

where α represents the angle (in radians) between the normal of the palm and the *Y*-axis of the sensor.

In the adaptive strategy, weights are allocated based on the calculation of confidence. The higher the confidence, the greater the weight assigned. Therefore, the calculation of weights is synchronized with confidence.

(14) weight=confidence

The data frame from the Leap Motion sensor included the palm’s normal vector, and α is calculated using the spatial vector method to measure the angle between the palm’s normal vector and the sensor’s *Y*-axis. Each sensor continuously updates the palm’s normal vector in real time. Simultaneously, at each time point, the palm’s normal vector detected by each sensor differs slightly. Consequently, this leads to slight variations in the α associated with each sensor. This difference also dynamically affects the confidence levels and weights.

In the Kalman filtering algorithm, the Kalman gain is typically calculated by balancing the prediction and measurement values to achieve the optimal state estimation. When multiple sensors or measurement sources are involved, the normalized weights can be used to adjust the contribution of each sensor or measurement to the Kalman gain, thereby influencing the state estimation of the Kalman filtering algorithm. This approach enables dynamic weight balancing of different sensor data, leading to more accurate and stable state estimation. This weight distribution also ensures that among the three sensors, even if two of them track incomplete or erroneous data, the tracking results remain more accurate.

#### 2.3.4. Safety Control Strategy Based on Pressure Sensor

**Figure 7:**
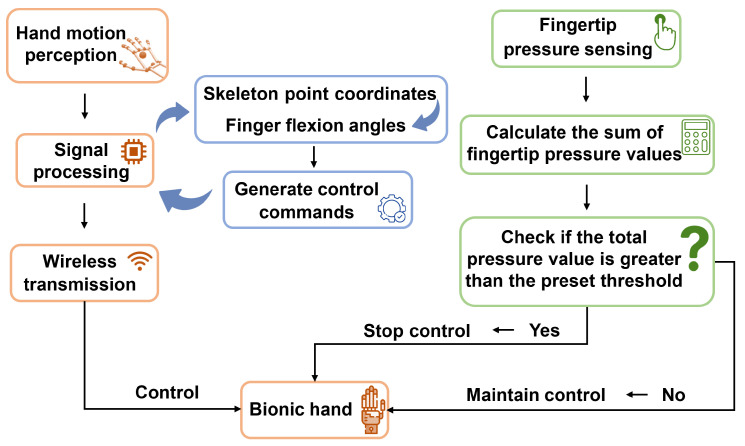
Algorithm flowchart of the safety control strategy based on pressure sensors, where, upon meeting the conditions after evaluation, it gains absolute control.

The six-degree-of-freedom bionic hand we used is equipped with pressure sensors at each fingertip. Taking a single fingertip as an example, there are 16 tactile sensors arranged in a 4 × 4 array. The force measurement range for a single point is 0 to 5 N. It employs resistive measurement methods to perceive the gripping force of the bionic hand. To calculate the pressure on the fingertips, we developed a method. The method mainly works by summing all the pressure values of the fingertip and comparing them with a preset pressure threshold. When the sum exceeds the threshold, the bionic hand will stop moving; Otherwise, normal movement will continue. Where the preset threshold is determined through a combination of experimentation and prior knowledge. The flow chart of the algorithm is shown in Figure 7, which mainly shows the overall structure of the algorithm. The specific calculation formula is as follows

(15) $$f=abs(sum\left(a_{1}+a_{2}+\cdots +a_{16}\right))$$

where *f* represents the pressure perceived by the fingertip and *a* represents the single point pressure value, there are 16 in total.

We developed this strategy to provide a safer way to control the bionic hand. In this way, when using the bionic hand to grasp fragile or soft objects, we can better control the grip force to avoid damaging or deforming the object. With this control, we can more accurately adjust the force of the bionic hand to meet the needs of different objects and provide more stable and reliable operation. This will improve the efficiency and safety of our work when handling vulnerable items.

## 3. Experiment and Demonstration

To validate the effectiveness and robustness of our proposed sensor fusion-based control strategy for a humanoid robotic arm and the arm motion tracking system we have developed, we conducted a series of experiments. The purpose of these experiments was to assess the performance and reliability of our control strategy and system in various scenarios. Firstly, we conducted real-time tracking experiments on a human arm using a depth camera. To enhance the quality of data collection, we employed a Kalman filtering algorithm to obtain smoother and more stable data. In the subsequent phases, we performed real-time hand motion tracking using multiple Leap Motion controllers, intentionally skipping the calibration step. By algorithmically fusing hand skeletal framework data, we validated the effectiveness of our proposed approach. To assess the robustness of our multi-sensor fusion system, we deliberately obstructed one of the sensors. This experiment was crucial in evaluating how well our system could adapt to unexpected environmental conditions and sensor failures. Finally, we verified the effectiveness of our safety control strategy based on fingertip pressure sensors for securely gripping fragile objects with the humanoid robotic arm.

Through these experiments, our objective was to comprehensively validate the performance of our proposed control strategy and system, ensuring their stability and reliability across diverse scenarios. This will provide strong support for the control and operation of humanoid robotic arms in practical applications, further advancing the development and application of robotics technology.

### 3.1. Arm Motion Tracking Experiment

#### 3.1.1. Experimental Content

**Figure 8:**
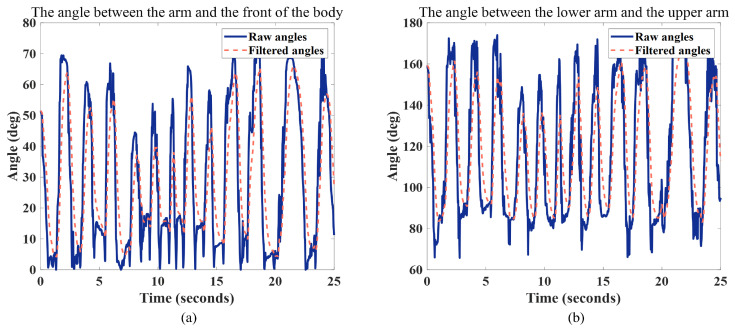
(**a**) The change curve of the angle between the arm and the front side of the body; (**b**) the change curve of the angle between the upper arm and the lower arm. Both have undergone Kalman filtering.

We employ the Kinect depth camera for extracting the skeletal information of the human arm. Subsequently, we compute the joint angles using the data from each joint point. Ultimately, we implement the kinematic mapping method to ensure accurate control over each joint in the anthropomorphic arm. Throughout this process, we select two noteworthy angles and apply Kalman filtering to them to enhance the smoothness and reliability of the data. The detailed curve depicting the changes in angles has been illustrated in Figure 8.

We selected two angles (from when the human body does reciprocating motion) and carried out filtering processing. The first angle is the angle between the arm and the front side of the body, and it partially reflects the arm’s position and orientation relative to the body. This angle can be used to assess the arm’s posture and position, especially in robot control, where understanding the arm’s relative position to the body is crucial for avoiding collisions and ensuring the robot performs the required tasks. The second angle is the angle between the upper arm and the lower arm, and it reflects the degree of bending at the arm’s joints. This angle can be used to evaluate the joint movements and bending degree of the arm, which is essential for controlling the motion of the robot arm and executing specific tasks. The experimental results revealed that the raw angle data exhibited noticeable fluctuations. However, after undergoing Kalman filtering, the angle data became significantly smoother. This smoother angle data provides operators with more precise and controllable arm movements, reducing unnecessary oscillations and instability. It allows us to control the humanoid robotic arm more accurately in performing various tasks. This has significant applications in various fields, including medical surgery, remote control in hazardous environments, and assembly tasks.

#### 3.1.2. Algorithm Comparison

To validate the superior performance of the Kalman filtering, we employed four common filtering methods: mean filtering, median filtering, low-pass filtering, and Kalman filtering. These methods were applied to process the data from two angles. Subsequently, we computed the root mean square error (RMSE) for each filtering method when processing the data. The formula for calculating RMSE is represented by Equation (16) [54].

(16) $$RMSE=\sqrt{\frac{1}{n}\sum_{i=1}^{n}{\left(y_{i}-{\hat{y}}_{i}\right)}^{2}}$$

where RMSE is a variable used to measure the error between the estimated value and the actual observation value, *n* is the total number of samples, $y_{i}$ is the *i*th observation value, and ${\hat{y}}_{i}$ is the *i*th estimate.

**Figure 9:**
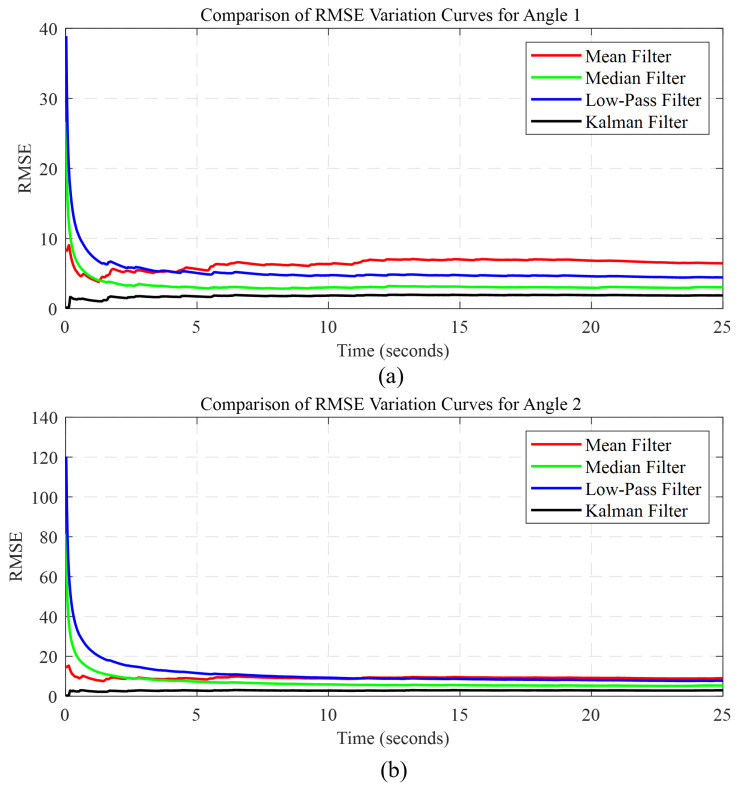
(**a**) Comparison of RMSE variations for the angle between the arm and the front side of the body; (**b**) comparison of RMSE variations for the angle between the upper arm and the lower arm.

For a clearer performance comparison, we generated comparative RMSE change curves for both angles, as shown in Figure 9. These visual representations provide an intuitive display of the RMSE variations for the different filtering methods.

Furthermore, we summarized the RMSE means for each filtering method, which are included in the RMSE mean comparison table (Table 1). A smaller RMSE indicates a lower level of error between the model or estimation and the actual data, signifying a higher level of fitting.

Through comparison, it is evident that the Kalman filter exhibits superior performance, and the successful application of the Kalman filtering algorithm not only enhances the system’s stability but also offers greater precision and control for remote humanoid robotic arm operation.

#### 3.1.3. Comparison of Trajectory Smoothness Metrics

In order to verify the effectiveness of the algorithm on angle filtering, we introduce trajectory smoothness as a quantitative index to compare the smoothness of the original data and the filtered data. The calculation of trajectory smoothness depends on the second derivative of the data, the average of its squares. This indicator reflects the smoothness of the data over the entire motion trajectory, i.e., the degree to which the data change. The specific calculation formula is as follows:

(17) $$Smoothness=\frac{1}{N}\sum_{i=1}^{N}{\left(\frac{d^{2}\theta }{dt^{2}}\right)}^{2}$$

where *N* is the number of samples, $\frac{d^{2}\theta }{dt^{2}}$ represents the second derivative of angle θ with respect to time *t*, and the average of the sum of squares represents the average degree of change in acceleration, so a smaller smoothness value means that the data changes more smoothly.

**Figure 10:**
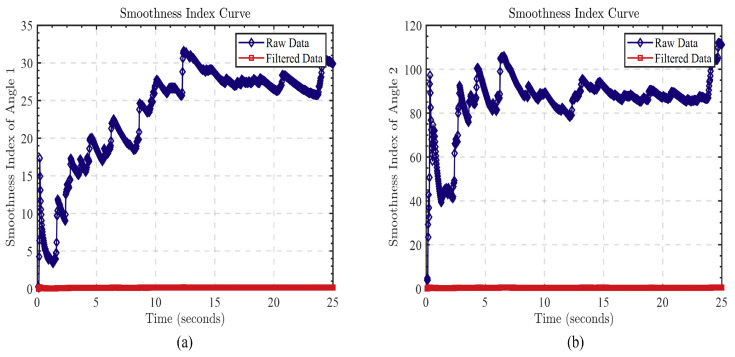
(**a**) The curve depicting the change in the smoothness index of the angle between the arm and the front side of the body; (**b**) the curve illustrating the change in the smoothness index of the angle between the upper arm and the lower arm.

In order to present the comparison results in a clearer way, we plotted the curve of the smoothness index over time, as shown in Figure 10.

From Figure 10, we can clearly observe that the smoothness values of the original data are significantly higher than those of the filtered data. This indicates that the trajectory data processed by the algorithm presents a more significant smoothness.

### 3.2. Hand Tracking Experiment under Multi-Sensor Framework

**Figure 11:**
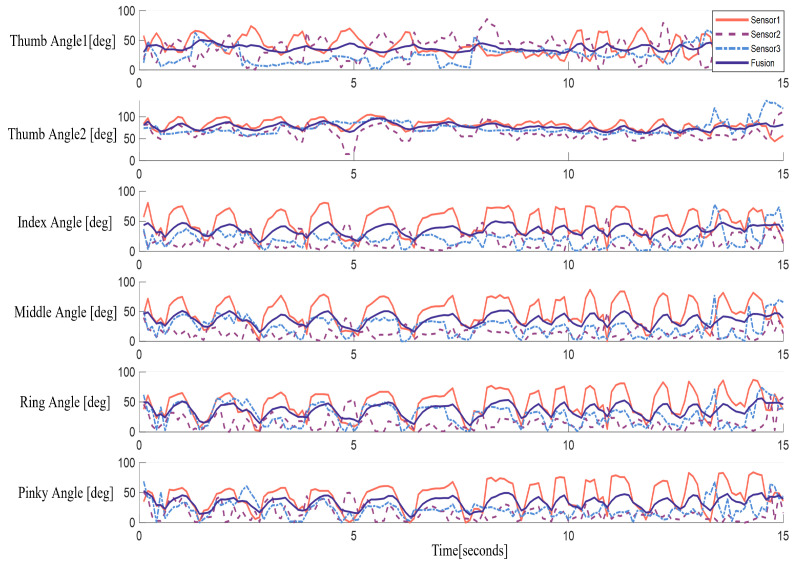
Experimental results of hand tracking under multi-sensor framework.

We placed the Leap Motion controller on a slidable track to change its spatial position and did not calibrate it. Since the bone point data are converted and calculated in the hand coordinate system, it has nothing to do with the spatial position of the controller. As shown in Figure 11, this system calculates the bending angle of each finger, and fuses the data from multiple sensors through an algorithm, so that the calculated angle is more stable and reliable. Through this fusion, we were able to obtain more accurate information on the bending angle of the finger, improving the performance and accuracy of the system.

In this experiment, because the Leap Motion controller did not need to be calibrated, it was free to adjust its position as needed for optimal hand tracking. In Figure 11, the data change from six angles over 15 s are shown. The bending angle of the thumb and the angle of abduction and adduction were calculated separately. It can be observed that due to the different spatial positions of the three Leap Motion controllers, there will be certain differences in the angles captured by different sensors when performing different hand movements. This difference is mainly due to the occlusion of the finger or palm within the field of view of a certain sensor during hand movement. As a result, the calculated angle data are not entirely reliable.

To solve this problem, we adopted a method of calculating confidence, assigning a weight to each sensor. By analyzing the spatial position relationship between the hand and the sensor, we can assess how reliably each sensor captures hand movements. Depending on the confidence, we can assign different weights to the data of each sensor and use the Kalman filtering algorithm to fuse the data.

Through data fusion, we can obtain a more reliable and stable angle value for controlling the bionic hand. This ensures that the Leap Motion controller can provide accurate and reliable hand posture data for different positions and movements, enabling a more accurate hand control experience.

**Figure 12:**
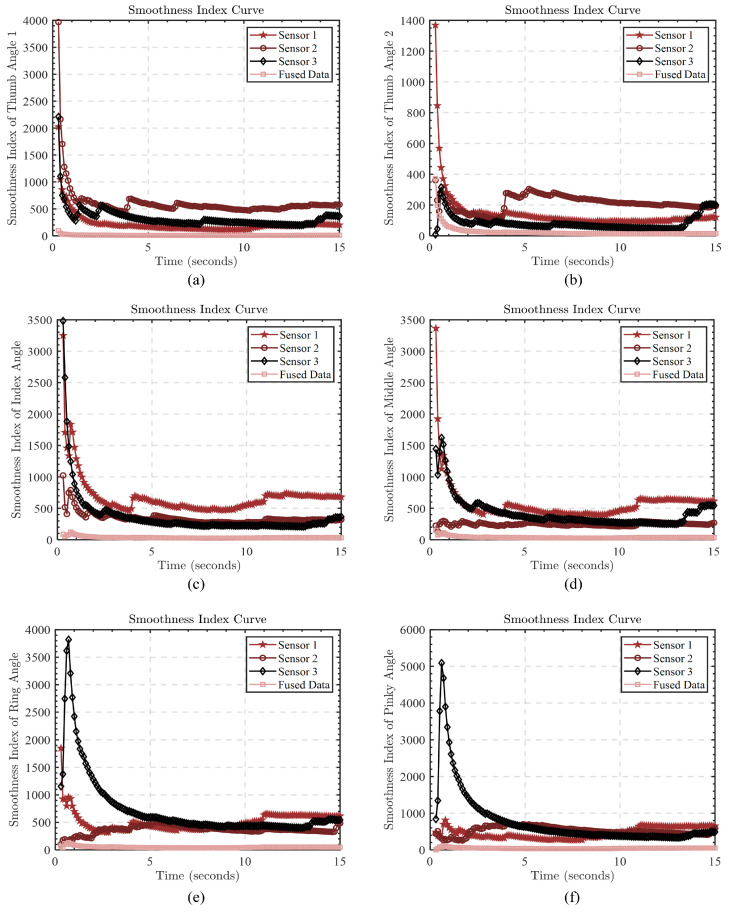
The trajectory smoothness index change curves of finger bending angles and thumb abduction-adduction angles over time. (**a**) The change curve of the thumb bending angle trajectory smoothness index; (**b**) the change curve of the thumb abduction-adduction angle trajectory smoothness index; (**c**) the change curve of the index finger bending angle trajectory smoothness index; (**d**) the change curve of the middle finger bending angle trajectory smoothness index; (**e**) the change curve of the ring finger bending angle trajectory smoothness index; (**f**) the change curve of the little finger bending angle trajectory smoothness index.

In order to verify the effectiveness and superiority of the fusion algorithm, we also adopted the quantitative index of trajectory smoothness to compare the difference in smoothness between the data trajectory obtained by a single sensor and the data trajectory after the algorithm fusion. In order to show the comparison results more clearly, we also drew the curves of different sensors and the smoothness index after algorithm fusion over time (as shown in Figure 12). In these curves, the smaller the smoothness value, the smoother the data changes.

It can be clearly seen from the figure that the trajectory smoothness value of the data processed by the fusion algorithm is significantly lower than that of other single sensors. This clearly shows that fusion algorithms play a significant role in improving data smoothness and make a positive contribution to the stability of the data.

### 3.3. Occlusion Experiment

**Figure 13:**
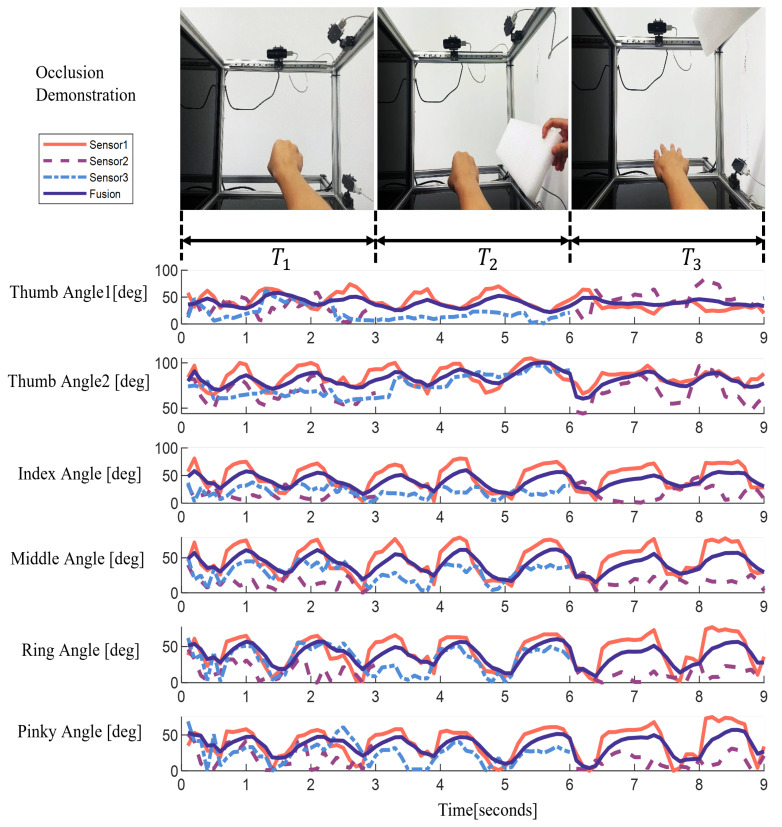
Occlusion experiment and results.

On the basis of the previous experiment, we further verified the robustness of this system in tracking the skeletal framework of the hand. By blocking either sensor (as shown in Figure 13), we can still obtain a stable angular data output. This result fully proves the reliability and robustness of the fusion strategy proposed in this paper.

In this experiment, we occluded different Leap Motion controllers at different time periods, and simultaneously collected data from each Leap Motion controller for angle calculation. As can be clearly seen from Figure 13, when one of the sensors is blocked, the blocked sensor no longer outputs the corresponding angle information during this period of time. However, this does not affect the hand-tracking capabilities of the other two sensors.

We adopt the Kalman filter fusion algorithm to solve the occlusion problem. By fusing data from multiple sensors, the algorithm can reduce noise and errors and improve the accuracy and stability of data. Even during the time period when occlusion occurs, we can still obtain a stable and reliable angle output. This is because the Kalman filter algorithm can estimate and predict the unknown data according to the existing data and system model, so as to make up for the data loss caused by the occlusion sensor. By using the data fusion algorithm, we can still obtain reliable hand posture data during the time period when occlusion occurs, so as to achieve accurate control of the bionic hand. This design ensures that in practical applications, even if a sensor fails, the user can continue to perform hand operations without being affected.

The redundancy design and the application of the data fusion algorithm effectively improve the reliability and stability of the system, and provide a better user experience. With or without occlusion, our system provides accurate hand tracking and angle calculations, ensuring that users can freely control the bionic hand for a variety of complex movements.

### 3.4. Safety Control Experiment Based on Pressure Sensor

**Figure 14:**
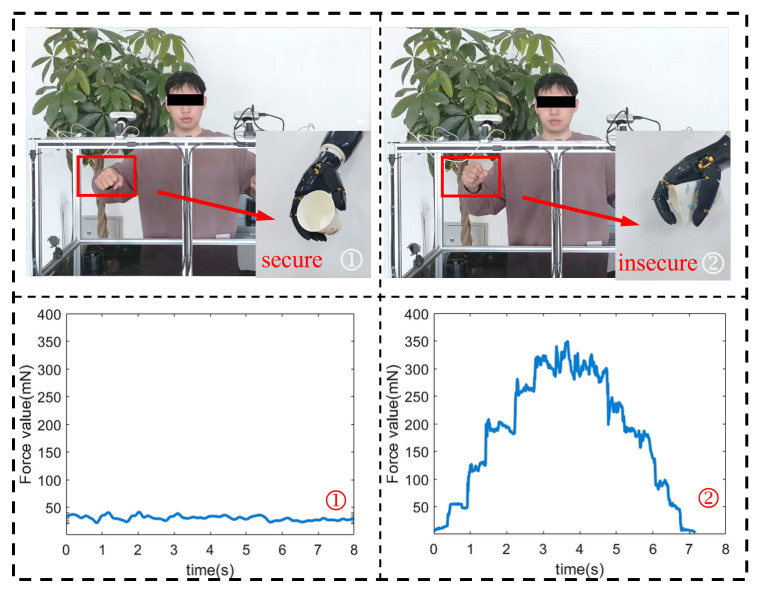
Comparison experiment between force control and non-force control scenarios. The numbers 1 and 2 represent Scenes 1 and 2, respectively.

In this experiment, we used the bionic hand to grasp the paper cup, respectively, when the force control was opened and when the force control was not opened, as shown in Figure 14. First of all, when we turn on the force control, the bionic hand can grasp the paper cup stably, and there is no large deformation of the paper cup in the process of grasping. However, when we do not turn on the force control, the paper cup has a large deformation in the process of grasping the paper cup by the bionic hand.

In Figure 14, we compared the change curve of the sum of the pressure values of the middle finger in the force-controlled and non-force-controlled scenarios. In scenario 1, we turned on force control mode. When the total pressure value exceeds the preset threshold (set to 35 mN in this experiment, it is predetermined based on experimental and prior knowledge), the bionic hand stops moving and the total pressure value of the pressure sensor in the middle finger tends to stabilize. This is because the force control mode can control the movement of the bionic hand according to a preset threshold, when the threshold is reached, even if the human hand further grips, the bionic hand will not further apply external forces to avoid over-squeezing the object. However, in scenario 2, since the force control mode is not turned on, the total pressure value of the pressure sensor continues to increase until the paper cup is severely deformed. The contact area between the pressure sensor and the paper cup is reduced, and the pressure value will gradually decrease. In this case, once the human hand is further grasped, the bionic hand will also apply external forces to the paper cup, causing excessive deformation of the paper cup.

By comparing the two scenes, we can see that the open force control mode can improve the holding stability of the bionic hand under the same condition of teleoperation. By setting the right threshold, we can ensure that even when the hand is making a fist, the bionic hand does not apply excessive force, thus avoiding damage and breakage of the object. This is especially important for grasping fragile and deformable objects, as it can provide a safer and more reliable control method that protects the integrity of the object. Therefore, the application of force control mode provides an important guarantee for the practical application of bionic hands. By reasonably setting the threshold and monitoring the pressure value of the bionic fingertip, we can realize the accurate control of the bionic hand, improve the stability of the grip, and thus expand the applicability and reliability of the bionic hand in various application scenarios.

## 4. Discussion

### 4.1. Overcoming Workspace Limitations for Humanoid Dual-Arm Robots

While the integration of anthropomorphic robotic hands into two-armed robots has undoubtedly enhanced their ability to achieve human-like control, a fundamental challenge remains largely unaddressed—confined workspaces. Unlike humans, this robotic system is anchored to a fixed base, greatly limiting its coverage and operational flexibility. This inherent limitation poses a significant barrier to realizing the full potential of humanoid dual-arm robots in numerous applications.

The fixed base of humanoid two-arm robots limits their ability to access certain areas, especially those located in hard-to-reach or confined Spaces. In areas critical to mobility and accessibility, such as construction, search and rescue, and manufacturing, current humanoid robot designs do not meet the needs of real-world scenarios. In addition, tasks that involve reaching high or low objects, navigating through cluttered environments, or accessing remote locations are severely hampered by this inherent limitation.

To address this critical challenge, our future work will focus on enhancing the capabilities of humanoid two-arm robots by introducing mobile mounts and telescopic mechanisms. The addition of a mobile chassis will allow robots to travel through their surroundings, expanding their coverage and adaptability. This mobility is critical in scenarios where new areas must be repositioned or explored, such as disaster response tasks, logistics, and outdoor construction. In addition, the combination of telescopic or lifting mechanisms, such as lifting thrusters or telescopic arms, will allow the robot to overcome the limitations associated with height. By extending their reach vertically, humanoid two-armed robots can reach higher objects, extending their usefulness to a wide range of applications, including warehouse automation, maintenance tasks, and infrastructure inspections.

In conclusion, while the integration of anthropomorphic hands into two-armed robots represents a major leap towards human-like control, the fundamental challenge of workspace constraints remains a huge obstacle. Combining mobile bases and telescopic mechanisms in future research work is expected to remove these limitations and open up new horizons for the deployment of humanoid two-arm robots in various fields. These advances will not only enhance the adaptability and versatility of these robotic systems, but will also expand their potential to handle complex real-world tasks with greater efficiency and effectiveness.

### 4.2. Overcoming the Limitations of Depth Visual Tracking

However, despite the remarkable progress we have made in the control of two-armed robots, current depth vision tracking technology still imposes certain limitations on the free movement of humans in some situations. Depth vision tracking typically requires the operator to remain within the camera’s field of view and is susceptible to interference from environmental factors, such as bright light, obstacles, and changes in perspective. These limitations can affect the operator’s degree of freedom and flexibility when controlling a two-armed robot, especially in situations that require movement or access to complex environments.

To address this challenge, our future work will focus on developing wearable device-based anthropomorphic motion control technologies. This technology will give operators a more natural, free, and intuitive way to control humanoid two-arm robots without the constraints of depth vision tracking. Wearable devices can be equipped with a variety of sensors and other sensing technologies, which can obtain real-time information about the movement of the operator, and maintain control of the robot no matter where they are.

This wearable device-based anthropomorphic motion control will not only increase the operator’s freedom but will also give them greater mobility, allowing them to comfortably control two-armed robots in a variety of environments. This is especially important for tasks that require the operation of robots in complex, unrestricted environments, such as emergency rescue, construction, and other fields. Wearables will become an extension tool for operators, allowing them to move away from the traditional console and closer to the actual operation scenario, thus improving the efficiency and safety of the task.

In summary, although we have made significant progress in depth vision tracking and control of dual-arm robots, the limitations of depth vision tracking remain and remain a challenge for expanding the practical application of dual-arm robots. Therefore, future work will focus on developing wearable device-based anthropomorphic motion control technologies to improve the operator’s freedom and the flexibility of robot control. This will lay the foundation for achieving more extensive and natural human-machine collaboration in a variety of applications, driving robotics forward to better serve human needs.

### 4.3. Expansion of Future Work

In this study, we employed a remote control approach to operate the robot. In comparison to traditional control methods, this remote control method offers several notable advantages.

Firstly, remote control overcomes the limitations of geographic distance. Traditional control methods typically require the operator to be physically present in the same location as the robot, whereas remote control allows operators to control the robot from a distance at any time. This has significant implications in various application scenarios, such as emergency rescue operations, tasks in hazardous environments, and remote sensing control. Furthermore, remote control enhances the level of safety. Traditional control methods may necessitate the on-site presence of the operator, exposing them to potential risks or hazards. Remote control helps mitigate these risks, enabling operators to maintain a safe distance.

However, it is important to note that remote control methods come with certain challenges and limitations. Network latency and instability can potentially affect real-time performance and control accuracy. Additionally, the reliability of sensors, communication devices, and algorithms is of utmost importance in remote control applications. In our future work, apart from exploring the potential applications of other sensors, such as wearable devices, we plan to investigate various communication frameworks and fusion algorithms. These communication frameworks may include DDS (data distribution service) and ZeroMQ, among others. Furthermore, we will delve into fusion algorithms better suited for nonlinear systems, such as extended Kalman filtering and unscented Kalman filtering, to further enhance the effectiveness and applicability of remote control.

In our force control safety experiment, we have implemented a threshold mechanism to ensure the safety of the bionic hand. If the force exerted by the bionic hand exceeds a predefined threshold, the system automatically halts the movement of the bionic hand. However, it is important to emphasize that the setting of this threshold is based on practical considerations and is not directly compared to human electromyography (EMG) signals or actual human output.

In future work, we plan to conduct a more comprehensive analysis by comparing the force output of the bionic hand with EMG signals from the human body or the forces exerted by the human body. This comparison will help us gain a better understanding of the relationship between the mechanical system’s force output and the natural capabilities of the human body, ensuring that the mechanical system’s force levels remain within a reasonable and safe range. Furthermore, in addition to capturing the actual output of the human body, we also plan to generate the actual motion trajectories of the robotic arm. This will allow us to compare the motion of the robotic arm with the actual human motion, aiding in a more comprehensive evaluation of our system’s performance.

As for the source of the experimental results, the experimental results of this study were obtained based on a single individual. We carefully monitored and recorded the data of a single individual in the experiment, and analyzed and discussed based on these data. In future work, we plan to expand the sample size and conduct statistical analyses to include more individuals to more fully understand potential individual differences and take appropriate approaches to compare or compensate. This will help to further verify the reliability and universality of the experimental results.

## 5. Conclusions

In this paper, a humanoid arm control strategy based on sensor fusion is proposed to achieve accurate control and humanoid expression of anthropomorphic arm movement. The control strategy uses the combination of depth camera and Leap Motion controller to sense the motion of the human arm and capture the gesture of the hand, thus building a complete human arm motion tracking system. First, the depth camera is used to sense the movement of a person’s arm. By extracting the skeleton information of the human arm, we can obtain the spatial position coordinates of the joint points of the human arm, and then calculate the joint angles through the algorithm, so as to map the joint servo of the humanoid arm to realize the control of the humanoid arm. The Leap Motion controller, meanwhile, is used to capture the movements of a person’s hands. By calculating the flexion angle of the finger, we can accurately express the gesture of the finger, so as to realize the anthropomorphic control of the arm movement. In order to improve the robustness and stability of the tracking system, the Kalman filter algorithm is used to process the perceived data. The algorithm can effectively eliminate the noise and uncertainty in the data, thus improving the accuracy and stability of the tracking system.

To verify the effectiveness of the proposed control strategy, we conducted a series of experiments. Firstly, we carried out experiments on arm and hand motion tracking to verify the accuracy and reliability of the system. Secondly, we conduct occlusion experiments to simulate tracking in complex environments. The experimental results show that the proposed control strategy is effective and robust. Finally, we conducted a comparison experiment between force-controlled and non-force-controlled scenarios. The experimental results show that the anthropomorphic control of the humanoid arm in the force control mode can improve grip stability, making it more suitable for grasping fragile and deformable objects. This provides a more secure and reliable control method for the humanoid arm in practical application.

## Figures and Tables

**Table 1:** Comparison of mean RMSE.

	Mean	Median	Low-Pass	Kalman
Angle 1	18.428	14.521	12.484	11.227
Angle 2	54.129	41.968	35.621	31.539

## Data Availability

Data are contained within the article.
